# Relationship between hearing thresholds and cognitive function in hearing aid non-users and long-term users post-midlife

**DOI:** 10.1038/s41514-025-00203-6

**Published:** 2025-02-24

**Authors:** Takanori Nishiyama, Tomomi Kimizuka, Chinatsu Kataoka, Mami Tazoe, Yasunori Sato, Makoto Hosoya, Marie N. Shimanuki, Takeshi Wakabayashi, Masafumi Ueno, Hiroyuki Ozawa, Naoki Oishi

**Affiliations:** 1 Department of Otolaryngology-Head and Neck Surgery, Keio University School of Medicine, Tokyo, Japan; 2 Otology and Audiology Center, Keio University Hospital, Tokyo, Japan; 3 Department of Preventive Medicine and Public Health, Keio University School of Medicine, Tokyo, Japan

**Keywords:** Disease prevention, Risk factors, Auditory system, Cognitive ageing

## Abstract

The extent of hearing loss requiring hearing aid (HA) to prevent cognitive decline is unclear; we assessed this post-midlife along with the relationship between hearing thresholds and cognitive function in those who had never used HA (non-users) or used HAs for >3 years (long-term users). This study comprised 117 individuals ≥55 years with an average hearing threshold of ≥25 dB HL in their better ear and 55 of the non-users and 62 of the long-term users. The Mini-Mental State Examination, the Symbol Digit Modalities Test (SDMT), and pure-tone and sound-field audiometry were assessed. Mean ± SD hearing levels of the non-user and long-term user group were 40.83 ± 8.16 and 51.13 ± 14.80 dB HL. Non-users showed a significant association (*P* = 0.01) between the hearing thresholds and SDMT scores, with a cutoff value of above 38.75 dB HL identified as affecting cognitive function. There were no significant associations for long-term users.

## Introduction

Recent research suggests that hearing loss is the largest potentially modifiable risk factor for dementia in midlife^1–3^ and is independently associated with accelerated cognitive decline and incident cognitive impairment in community-dwelling older adults^4^. Therefore, hearing loss interventions as preventive measures against dementia have gained much attention. Age-related hearing loss is the most common cause of hearing loss and typically manifests as bilateral sensorineural hearing loss, which predominantly results from inner ear cell damage^5^. Mammalian inner ear cells do not regenerate once damaged^6^; therefore, hearing aids or cochlear implants play a central role in treating sensorineural hearing loss. Age itself is also associated with cognitive decline^7^. Several studies on the impact of hearing aids and cochlear implants on cognitive function have indicated that their use may correlate with a reduction of the incidence of dementia^8–17^. However, the existing studies had certain limitations. First, many studies evaluated the effects of hearing aids over a follow-up period of only 6 months^8,11,18^. Considering that patients require several months to adapt to hearing aids^19^, we believe that a 6-month observation period is too short to determine the influence of hearing aids on cognitive function. Second, since many of the previous studies do not mention any details regarding adjustment to hearing aids^8–10,13,14,16,18^, some patients may not have adjusted well to hearing aids, despite the overall similarity in their usage patterns. The potential lack of benefit for patients who do not adjust well to hearing aid use may not have been considered in these previous studies. Similarly, studies using large databases have not been able to determine some patient variables, including adjustment to hearing aids^13,14^. Furthermore, dementia is expected to be prevented when hearing loss interventions are implemented during midlife, yet studies have predominantly focused on older adults^8,10–12,16,18^. Recently, Lin et al.^16^ reported the results of a 3-year randomized controlled trial on the effect of hearing aids on 977 participants with hearing impairment and compared a hearing aid intervention group and a health education group that did not use hearing aids. They suggested that a hearing intervention might reduce cognitive changes over a period of 3 years in populations of older adults at increased risk for cognitive decline. However, all participants were older than 70 years, and details related to the adjustment of their hearing aids were unclear.

Few studies have investigated the degree of hearing loss that may result in cognitive decline if left untreated^20^. Exploring this topic could help encourage older adults to use hearing aids, thereby improving hearing aid underutilization. We believe that elucidating this issue is also important for understanding the level of hearing aid amplification required to exert a preventive effect on cognitive decline.

The aim of this study was to determine the cutoff hearing threshold values that may affect cognitive function in people aged ≥55 years with bilateral average hearing thresholds of ≥25 dB HL at four frequencies (500, 1000, 2000, and 3000 Hz). We hypothesized that background factors differ between patients who have never used hearing aids and those who have used hearing aids for a long time. Therefore, we assessed the two groups to evaluate the relationship between hearing thresholds, hearing aid use, and cognitive function.

## Results

### Participant characteristics

Of the 580 patients who met the hearing criteria (average pure-tone audiometry hearing [PTA-average] thresholds of >25 dB HL [degree of hearing loss in decibels] at four frequencies [500, 1000, 2000, and 3000 Hz] bilaterally), 129 consented to cognitive function testing. After excluding 12 patients with a history of intracranial disease or dementia, 117 patients were included in this study (58 [50%] men; mean [standard deviation (SD)] age 75 [8] years; mean [SD] PTA-average threshold in the better-hearing ear 46.34 [13.19] dB HL). Among the 117 patients, 55 were included in the non-user group and 62 in the long-term user group. A comparison of background factors between the two groups revealed that the PTA-average threshold of the long-term user group was significantly higher than that of the non-user group (*P* < 0.001); however, there were no other significant differences, including cognitive function test results (Table 1). The age ranges of the non-user and the long-term user groups were 55–93 and 55–89 years old, respectively. The average (SD) daily use of hearing aids was 11.0 (5.4) h.

**Table 1:** Comparison of clinical characteristics of patients in the non-user and long-term user groups

Characteristic	Non-user group, mean (SD)	Long-term user group, mean (SD)	*P*-value
Participants, *n*	55	62	NA
Age, yrs	76 (8)	74 (8)	0.13^a^
Male sex, *n* (%)	24 (44)	35 (56)	0.17^b^
PTA-average threshold, dB HL	40.83 (8.16)	51.13 (14.80)	<0.001^a^
SF-average threshold, dB HL	NA	35.04 (9.32)	NA
MMSE-J	28 (2)	28 (2)	0.40^a^
MMSE-J ≤ 27, No. (%)	13 (24)	12 (20)	0.57^b^
MMSE-J < 24, No. (%)	2 (4)	2 (3)	0.90^b^
SDMT, %	39 (10)	42 (10)	0.14^a^

*dB HL* degree of hearing loss in decibels, *MMSE-J* Japanese version of the Mini-Mental State Examination, *NA* not applicable, *PTA* pure-tone audiometry, *SD* standard deviation, *SDMT* Symbol Digit Modalities Test, *SF* sound-field audiometry.

^a^Statistical analysis is performed using the Mann–Whitney *U* test.

^b^Statistical analysis is performed using the *χ*^2^ test.

### Assessment of hearing thresholds, hearing aid usage time, cognitive function, and age

**Fig. 1:**
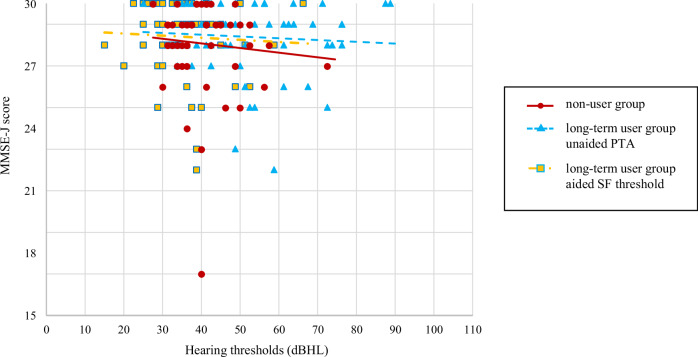
Relationship between hearing thresholds and MMSE-J. PTA-average for non-users (dots), PTA-average (triangles), and SF-average (squares) for long-term users, and the approximate straight line for each. dB HL degree of hearing loss in decibels, MMSE-J Japanese version of the Mini-Mental State Examination, PTA pure-tone audiometry, SF sound-field.

**Fig. 2:**
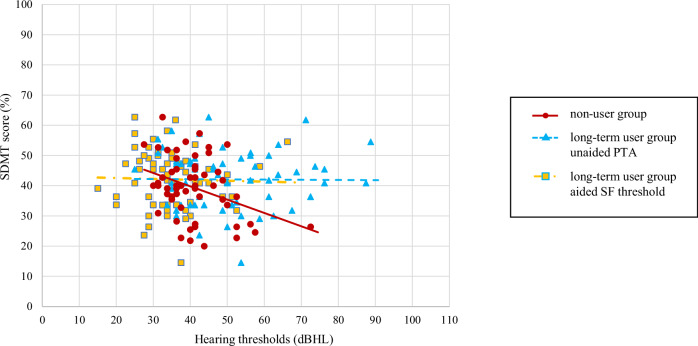
Relationship between hearing thresholds and SDMT. PTA-average for non-users (dots), PTA-average (triangles), and SF-average (squares) for long-term users, and the approximate straight line for each. dB HL degree of hearing loss in decibels, PTA pure-tone audiometry, SDMT Symbol Digit Modalities Test, SF sound-field.

In both groups, no significant correlation was found between Japanese version of the Mini-Mental State Examination (MMSE-J) scores and PTA-average threshold (non-user group: *ρ* = −0.030; *P* = 0.83 and long-term user group: *ρ* = −0.054; *P* = 0.67), whereas there was a significant correlation between Symbol Digit Modalities Test (SDMT) scores and PTA-average threshold in the non-user group (*ρ* = −0.27; *P* = 0.047). In the long-term user group, the PTA-average threshold showed no significant correlation with SDMT scores (*ρ* = −0.076; *P* = 0.56), and the sound-field (SF) -average threshold showed no significant correlation between MMSE-J scores (*ρ* = −0.050; *P* = 0.70) and SDMT scores (*ρ* = −0.11; *P* = 0.39) (Figs. 1 and 2). In the long-term user group, there was no significant correlation between hearing aid usage time and MMSE-J scores (*ρ* = −0.020; *P* = 0.88) or SDMT scores (*ρ* = 0.084; *P* = 0.52). Furthermore, no significant correlation was found between hearing aid usage time and aided SF-average threshold (*ρ* = −0.006; *P* = 0.96).

In the non-user group, there was no significant correlation between age and PTA-average threshold (*ρ* = −0.26; *P* = 0.060). In the long-term user group, age showed no significant correlation with the PTA-average threshold (*ρ* = −0.031; *P* = 0.81) or SF-average threshold (*ρ* = 0.20; *P* = 0.13). Age was significantly correlated with MMSE-J (non-user group: *ρ* = −0.31; *P* = 0.024 and long-term user group: *ρ* = −0.47; *P* < 0.001) and SDMT scores (non-user group: *ρ* = −0.61; *P* < 0.001 and long-term user group: *ρ* = −0.70; *P* < 0.001) in both groups.

### Hearing threshold cutoff values for cognitive decline

**Fig. 3:**
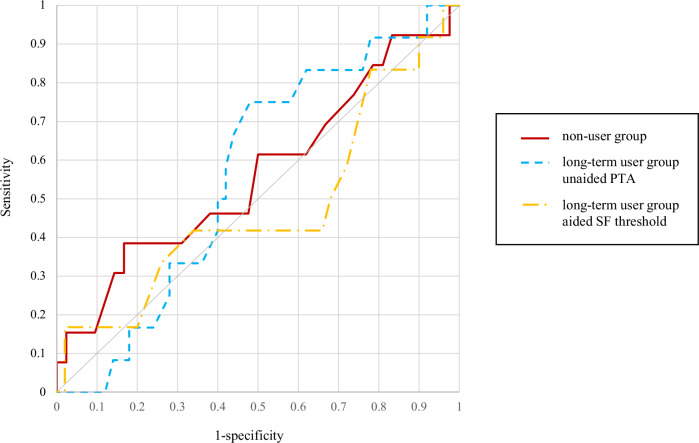
ROC analysis of hearing thresholds and MMSE-J. MMSE-J Japanese version of the Mini-Mental State Examination, PTA pure-tone audiometry, ROC receiver operating characteristic, SF sound-field.

**Fig. 4:**
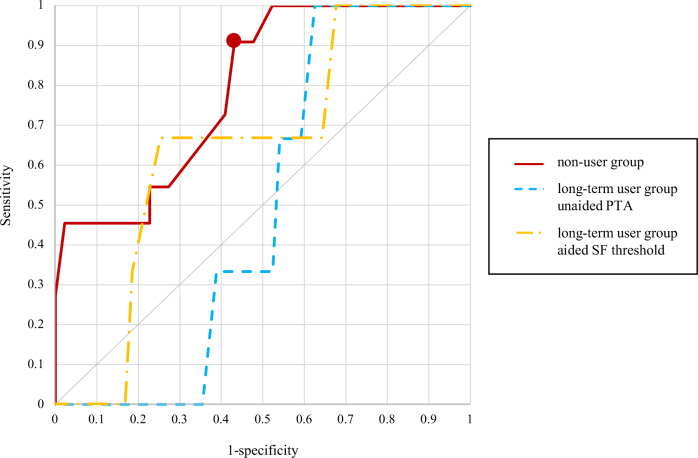
ROC analysis of hearing thresholds and SDMT. Dot: the point where the cutoff value is present. PTA pure-tone audiometry, ROC receiver operating characteristic, SDMT Symbol Digit Modalities Test, SF sound-field.

To estimate the hearing cutoff values that affect the cognitive score, receiver operating characteristic (ROC) analyses were performed for the PTA-average threshold of the non-user group, the PTA- and SF-average thresholds of the long-term user group, and each cognitive test result. In the non-user group, 13 of 55 patients had an MMSE-J total score of ≤27, and 10 of 55 patients had an SDMT score of ≤27.3%. A significant ROC curve could not be drawn between PTA-average threshold and MMSE-J scores (*P* = 0.50; area under the curve [AUC] = 0.57), although a significant ROC curve could be drawn between PTA-average threshold and SDMT scores (*P* < 0.0010; AUC = 0.79), and the cutoff value of the PTA-average threshold affecting the SDMT score was 38.75 dB HL in the non-user group. The sensitivity, specificity, positive predictive value (PPV), and negative predictive value (NPV) of this cutoff value were 0.91, 0.57, 0.34, and 0.96, respectively. In the long-term user group, 12 of 62 patients had an MMSE-J total score of ≤27, and three of 62 patients had an SDMT score of ≤27.3%. No significant ROC curves could be drawn between the unaided PTA-average threshold and MMSE-J scores (*P* = 0.43; AUC = 0.56) or SDMT scores (*P* = 0.95; AUC = 0.49) or between aided SF-average threshold and MMSE-J scores (*P* = 0.82; AUC = 0.48) or SDMT scores (*P* = 0.28; AUC = 0.65), and no significant cutoff values were determined (Figs. 3 and 4).

## Discussion

This study focused on determining the hearing threshold cutoff values that could affect cognitive function in people with hearing loss after midlife. We hypothesized that the hearing threshold cutoff would differ in patients who had never used hearing aids and in those who had used hearing aids for a long time. Therefore, we divided the participants into two groups and evaluated the relationship between hearing thresholds, use of hearing aids, and cognitive function. We found a significant negative correlation between the PTA-average threshold and SDMT scores in the non-user group and no significant correlation between the PTA- or SF-average threshold and cognitive function in the long-term user group. Although the long-term user group had a significantly greater PTA-average threshold than the non-user group, consistent with past findings^21,22^, cognitive function did not significantly differ between the two groups. These results suggest that in a group of individuals who have never used hearing aids, hearing loss is related to poorer cognitive scores. However, the negative relationship between hearing loss and cognitive function was mitigated by the long-term use of hearing aids. This result is consistent with previous findings related to hearing loss and dementia^1,2^ and further supports the use of hearing aids for preventing the progression of dementia^8–16,18^. Aging is also a factor associated with cognitive decline^7^, and in the present study, both age and MMSE-J and SDMT scores showed significant correlations. However, hearing thresholds showed an independent relationship with age, suggesting a different mechanism of cognitive decline than aging. Additionally, no significant correlation was found between MMSE-J scores and PTA- or SF-average thresholds in either group. Although MMSE-J is an effective tool for assessing cognitive function in individuals, the simplicity of the test renders it prone to ceiling effects^23–26^, which may make it difficult to detect differences within a given population. Future studies should consider employing more sensitive assessments that are less prone to ceiling effects, such as the Montreal Cognitive Assessment^23,26^.

Although the usage time was based on self-reporting and, therefore, was less reliable than that evaluated by data logging, no significant correlation was observed between hearing aid usage time and cognitive function. This suggests that it is not necessarily important to use hearing aids for long hours to prevent dementia. Instead, it underscores the importance of using them when needed based on an individual’s lifestyle. However, the mean duration of hearing aid use among the study participants was 11 h/day, which was longer than that reported previously^16^. In other words, participants had been using hearing aids for a relatively longer duration per day as a group, and the influence of patients who may not have benefited from hearing aids, such as those who had been using them for an extremely short duration per day, was relatively small. Moreover, no significant correlation was observed between the duration of hearing aid use and the SF-average threshold. Our results are consistent with those of studies reporting that hearing aid use is not only dependent on its efficacy^27^.

ROC analyses were performed on PTA- and SF-average threshold and cognitive function for the non-user and long-term user groups, classified by the presence of an MMSE score of ≤27 and an SDMT score of ≤27.3%. In the non-user group, the PTA-average threshold of 38.75 dB HL was a significant cutoff value for SDMT scores. In the long-term user group, there was no significant cutoff value for the PTA- or SF-average hearing threshold that could affect cognitive function, regardless of whether participants were aided or unaided. This suggests the need for active auditory compensation with hearing aids in participants with a PTA-average threshold of 38.75 dB HL or more for the prevention of cognitive decline. In this study, 29 of the 55 patients in the non-user group exceeded the PTA-average threshold of 38.75 dB HL, and eight of these 29 patients (27.6%) did not wish to use hearing aids. These participants had not been informed about the possible negative impact of hearing loss on cognitive function at the time of this study. We believe explaining that hearing aids may reduce the risk of cognitive impairment would help people with hearing loss make informed decisions about being fitted with hearing aids. In contrast, in the long-term user group, the cutoff values of the PTA- or SF-average thresholds associated with a risk of cognitive decline were not identified. As previously mentioned, the unaided hearing threshold (i.e., PTA-average threshold of the long-term user group) no longer correlated with cognitive function, possibly because the negative effects on cognitive function were eliminated by the long-term use of hearing aids. Regarding the aided hearing threshold (i.e., SF-average threshold of the long-term user group), 19 of 62 cases (30.6%) exceeded 38.75 dB HL, which was the cutoff value in the non-user group. Of the 43 cases with an aided hearing threshold of 38.75 dB HL or less and 19 cases with an aided hearing threshold exceeding 38.75 dB HL, seven (16.3%) and five (26.3%) participants, respectively, had mild cognitive impairment (MCI) or worse. A trend towards MCI or worse was observed in participants with an aided hearing threshold exceeding 38.75 dB HL, but due to the small number of participants in this study, the results of larger studies are eagerly awaited. If the aided hearing threshold exceeds 38.75 dB HL, and communication remains significantly impaired despite appropriate hearing aid fitting, other treatment options, such as cochlear implants, should be considered.

This study had some limitations. First, a type of selection bias was present. All participants were patients who visited a university hospital in an urban area and agreed to undergo cognitive function testing; therefore, they may have had higher income and a higher education level than patients with hearing impairments in rural areas, and they had a relatively high interest in their health. In addition, hearing aid usage time was evaluated through participant self-reporting, so bias cannot be ruled out. This was a cross-sectional study that did not assess changes in hearing thresholds or cognitive function over time in patients with and without hearing aids. Patients with hearing impairment and poor cognitive function might have difficulty continuing to use hearing aids for long periods; therefore, the long-term user group might be composed of patients with relatively high cognitive function. We used the average of the 500, 1000, 2000, and 3000 Hz hearing thresholds as the hearing level in this study. However, to account for age-related changes, if we used the average of the 500, 1000, 2000, and 4000 Hz hearing thresholds as the hearing level, the cutoff value could have been somewhat higher. In addition, the number of patients in each group was relatively small.

In conclusion, among patients older than 55 years who had hearing loss and had never used hearing aids, a significant negative correlation was found between hearing threshold and cognitive function, and a PTA-average threshold of 38.75 dB HL was the cutoff value for the possible risk of cognitive decline. However, in patients with hearing loss who had used hearing aids for more than three years, no significant correlation was observed between cognitive function and the cutoff values for hearing thresholds that could be risk factors for cognitive decline. The PTA-average threshold of ≥38.75 dB HL may be a risk factor for cognitive decline among hearing aid non-users who are in midlife and beyond. The long-term use of hearing aids may potentially reduce this risk.

## Methods

### Ethical considerations

This cross-sectional, prospective, cohort study was conducted in accordance with the Ethical Principles for Medical Research Involving Human Subjects expressed in the Declaration of Helsinki^28^ and was approved by the Institutional Review Board of Keio University School of Medicine (approval number 20200033). Verbal informed consent was obtained from all participants. Written informed consent was not obtained because it is not required by the Japan Ethical Guidelines for Medical and Health Research Involving Human Subjects^29^. Participants were also informed that they could decline participation at any time and that their data would be deleted after the study concluded. If the screening results indicated the presence of dementia, we recommended referral to a dementia treatment specialist within Keio University Hospital.

### Participants

Patients aged ≥55 years who visited Keio University Hospital between September 2022 and September 2023 were selected for the study. The inclusion criteria were: average PTA hearing thresholds of >25 dB HL at four frequencies (500, 1000, 2000, and 3000 Hz) bilaterally according to the American Academy of Otolaryngology-Head and Neck Surgery Foundation (AAO-HNSF) guideline^30^; no history of hearing aid use, or use of hearing aids for >3 years; and agreement to undergo cognitive function testing. We defined long-term hearing aid use in the current study as using hearing aids for >3 years. The rationale for this is that we considered that 6 months of hearing aid use was too short a period to habituate to hearing aids and influence cognitive function; we considered 3 years to be a sufficient period, similar to the observation period in the study by Lin et al.^16^. All participants were native Japanese. Patients with intracranial diseases, such as head trauma and brain tumors, and those with a previous diagnosis of dementia were excluded.

### Hearing assessment

Audiometric thresholds were measured by experienced audiometric technicians in a standard sound-attenuated booth using a commercially available audiometer (Model AA-H1; RION Co., Ltd., Tokyo, Japan). Air conduction PTA thresholds at eight frequencies (125, 250, 500, 1000, 2000, 3000, 4000, and 8000 Hz) were assessed bilaterally using over-ear headphones. The average hearing threshold values at 500, 1000, 2000, and 3000 Hz were used to calculate the PTA-average threshold. It is also common practice to consider 4000 Hz instead of 3000 Hz when evaluating average hearing thresholds. However, previous reports have indicated that amplification from hearing aids is difficult to obtain after 4000 Hz^31^ and that amplification in the high frequencies after 4000 Hz does not always produce good results for speech recognition^32,33^. We believe evaluating hearing thresholds at 3000 Hz is more important when considering hearing aid amplification. Therefore, we included 3000 Hz instead of 4000 Hz for assessing the average of the four frequencies. SF thresholds were measured to assess aided thresholds using warble tones presented from a loudspeaker placed 1 m from the participant at 0° azimuth to assess six frequencies (250, 500, 1000, 2000, 3000, and 4000 Hz). The average of 500, 1000, 2000, and 3000 Hz of aided thresholds was considered as the SF-average hearing threshold. Aided hearing thresholds for binaural hearing aid users were evaluated one ear at a time, and, if necessary, narrow band noise was presented to the other ear through headphones to prevent the influence of non-testing ears. If a hearing threshold of 500, 1000, 2000, or 3000 Hz exceeded 110 dB HL, 5 dB was added to the maximum presented sound pressure, serving as the adjusted hearing threshold. We defined the ear with the better PTA-average threshold as the better-hearing ear.

### Hearing aid adjustment

The participants were divided into two groups: those who had never used a hearing aid (non-user group) and those who had used hearing aids for >3 years (long-term user group). The long-term hearing aid users were fitted with hearing aids for 3 months before purchase, and gain adjustment was performed according to the Utsunomiya method^19^. After purchasing hearing aids, the patients visited the clinic approximately every 6 months for hearing follow-up and hearing aid maintenance. Daily hearing aid usage time was self-reported since not all the hearing aids had logging capabilities.

### Cognitive function assessment

Cognitive function was assessed using the MMSE-J and the SDMT in both groups. The MMSE-J is a 30-point questionnaire that can assess disorientation and short-term memory. A total score of ≥28 indicates no cognitive impairment, a score of 24–27 indicates MCI and a score of ≤23 indicates the presence of severe cognitive impairment^34^. The SDMT involves memorization of numbers and corresponding symbols. The SDMT is reported to be sensitive to very early changes in aspects of mental speed and attention in Alzheimer’s disease^35,36^. It assesses the number of accurate responses, wherein participants write symbols corresponding to the numbers given within a 90-s time frame. The SDMT score is divided by 110, which is the maximum number of questions, and expressed as a percentage^37^. In accordance with previous reports, an SDMT score of 27.3% was considered the cutoff, and findings below this were considered indicative of mild cognitive impairment^38,39^. Cognitive function tests were conducted in a private room by a trained psychologist to check reliable responses from the participants. These tests were administered orally, and we also provided participants with printed versions of the questions to ensure their complete understanding. Participants in the long-term user group were tested in an aided condition.

### Statistical analyses

We compared demographic variables (e.g., age, sex, PTA-average threshold, SF-average threshold, MMSE-J total score, number and proportion of MMSE-J total score <27 or 24, and SDMT score) between the two study groups using the Mann–Whitney *U* test for continuous variables and *χ*^2^ tests for categorical variables. The relationship between PTA-average threshold and cognitive scores and age in the non-user group, between PTA- and SF-average thresholds and cognitive test results and age, and between hearing aid usage time and cognitive test results or SF-average threshold in the long-term user group were examined using Spearman’s rank correlation coefficients. We assessed the diagnostic performance of hearing thresholds for cognitive decline (MMSE-J total score ≤27 or SDMT score ≤27.3%) using ROC curves. Analyses included sensitivity, specificity, PPV, NPV, and AUC. Subsequently, the Youden index, equal to the sensitivity + specificity – 1, was optimized from the ROC curve to determine the cutoff values for the PTA- and SF-average thresholds. All statistical analyses were performed using SPSS version 28 (IBM Corporation, Armonk, NY, USA), and a *P*-value of <0.05 was considered statistically significant.

## Data Availability

The data that support the findings of this study are available from the corresponding author upon reasonable request.

## References

[1] Livingston, G. et al. Dementia prevention, intervention, and care. *Lancet* **390**, 2673–2734 (2017). 28735855 10.1016/S0140-6736(17)31363-6

[2] Livingston, G. et al. Dementia prevention, intervention, and care: 2020 report of the Lancet Commission. *Lancet* **396**, 413–446 (2020). 32738937 10.1016/S0140-6736(20)30367-6 PMC7392084

[3] Livingston, G. et al. Dementia prevention, intervention, and care: 2024 report of the Lancet Standing Commission. *Lancet* **404**, 572–628 (2024). 39096926 10.1016/S0140-6736(24)01296-0

[4] Lin, F. R. et al. Hearing loss and cognitive decline in older adults. *JAMA Intern. Med.* **173**, 293–299 (2013). 23337978 10.1001/jamainternmed.2013.1868 PMC3869227

[5] Tawfik, K. O., Klepper, K., Saliba, J. & Friedman, R. A. Advances in understanding of presbycusis. *J. Neurosci. Res.* **98**, 1685–1697 (2020). 30950547 10.1002/jnr.24426

[6] Zhang, S. et al. Hair cell regeneration from inner ear progenitors in the mammalian cochlea. *Am. J. Stem Cells* **9**, 25–35 (2020). 32699655 PMC7364385

[7] Walsh, C. E. et al. Age profiles of cognitive decline and dementia in late life in the aging, demographics, and memory study. *J. Gerontol. B Psychol. Sci. Soc. Sci.* **77**, 1880–1891 (2022). 35171992 10.1093/geronb/gbac038 PMC9535777

[8] Mulrow, C. D. et al. Quality-of-life changes and hearing impairment. A randomized trial. *Ann. Intern. Med.* **113**, 188–194 (1990). 2197909 10.7326/0003-4819-113-3-188

[9] Deal, J. A. et al. Hearing impairment and cognitive decline: a pilot study conducted within the atherosclerosis risk in communities neurocognitive study. *Am. J. Epidemiol.* **181**, 680–690 (2015). 25841870 10.1093/aje/kwu333 PMC4408947

[10] Amieva, H. et al. Self-reported hearing loss, hearing aids, and cognitive decline in elderly adults: a 25-year study. *J. Am. Geriatr. Soc.* **63**, 2099–2104 (2015). 26480972 10.1111/jgs.13649

[11] Deal, J. A. et al. A randomized feasibility pilot trial of hearing treatment for reducing cognitive decline: results from the Aging and Cognitive Health Evaluation in Elders Pilot Study. *Alzheimers Dement.* **3**, 410–415 (2017). 10.1016/j.trci.2017.06.003 PMC5651440 29067347

[12] Sarant, J. et al. The effect of hearing aid use on cognition in older adults: can we delay decline or even improve cognitive function? *J. Clin. Med.* **9**, 254 (2020). 31963547 10.3390/jcm9010254 PMC7020090

[13] Naylor, G. et al. Dementia and hearing-aid use: a two-way street. *Age Ageing* **51**, afac266 (2022). 36571777 10.1093/ageing/afac266 PMC9792081

[14] Byun, H., Chung, J. H., Lee, S. H., Kim, E. M. & Kim, I. Dementia in a hearing-impaired population according to hearing aid use: a nationwide population-based study in Korea. *Ear Hear* **43**, 1661–1668 (2022). 35671072 10.1097/AUD.0000000000001249 PMC9592173

[15] Andries, E. et al. Evaluation of cognitive functioning before and after cochlear implantation in adults aged 55 years and older at risk for mild cognitive impairment. *JAMA Otolaryngol. Head Neck Surg.* **149**, 310–316 (2023). 36795400 10.1001/jamaoto.2022.5046 PMC9936380

[16] Lin, F. R. et al. Hearing intervention versus health education control to reduce cognitive decline in older adults with hearing loss in the USA (ACHIEVE): a multicentre, randomised controlled trial. *Lancet* **402**, 786–797 (2023). 37478886 10.1016/S0140-6736(23)01406-X PMC10529382

[17] Bucholc, M., Bauermeister, S., Kaur, D., McClean, P. L. & Todd, S. The impact of hearing impairment and hearing aid use on progression to mild cognitive impairment in cognitively healthy adults: an observational cohort study. *Alzheimers Dement.* **8**, e12248 (2022). 10.1002/trc2.12248 PMC8863441 35229022

[18] Uchida, Y. et al. A multi-institutional study of older hearing aids beginners-a prospective single-arm observation on executive function and social interaction. *J. Am. Med. Dir. Assoc.* **22**, 1168–1174 (2021). 33811828 10.1016/j.jamda.2021.02.035

[19] Shinden, S. et al. Effective sound therapy using a hearing aid and educational counseling in patients with chronic tinnitus. *Auris Nasus Larynx* **48**, 815–822 (2021). 33461856 10.1016/j.anl.2021.01.001

[20] Chien, W. & Lin, F. R. Prevalence of hearing aid use among older adults in the United States. *Arch. Intern. Med.* **172**, 292–293 (2012). 22332170 10.1001/archinternmed.2011.1408 PMC3564585

[21] An, Y. H. et al. Long-term effects of hearing aid use on auditory spectral discrimination and temporal envelope sensitivity and speech perception in noise. *J. Int. Adv. Otol.* **18**, 43–50 (2022). 35193845 10.5152/iao.2022.21228 PMC9449765

[22] Goel, A. R., Bruce, H. A., Williams, N. & Alexiades, G. Long-term effects of hearing aids on hearing ability in patients with sensorineural hearing loss. *J. Am. Acad. Audiol.* **32**, 374–378 (2021). 34082459 10.1055/s-0041-1731592

[23] Nasreddine, Z. S. et al. The Montreal Cognitive Assessment, MoCA: a brief screening tool for mild cognitive impairment. *J. Am. Geriatr. Soc.* **53**, 695–699 (2005). 15817019 10.1111/j.1532-5415.2005.53221.x

[24] Pendlebury, S. T. et al. Differences in cognitive profile between TIA, stroke and elderly memory research subjects: a comparison of the MMSE and MoCA. *Cerebrovasc. Dis.* **34**, 48–54 (2012). 22759627 10.1159/000338905

[25] Siqueira, G. S. A., Hagemann, P. M. S., Coelho, D. S., Santos, F. H. D. & Bertolucci, P. H. F. Can MoCA and MMSE be interchangeable cognitive screening tools? A systematic review. *Gerontologist* **59**, e743–e763 (2019). 30517634 10.1093/geront/gny126

[26] Jia, X. et al. A comparison of the Mini-Mental State Examination (MMSE) with the Montreal Cognitive Assessment (MoCA) for mild cognitive impairment screening in Chinese middle-aged and older population: a cross-sectional study. *BMC Psychiatry* **21**, 485 (2021). 34607584 10.1186/s12888-021-03495-6 PMC8489046

[27] Munson Klyn, N. A., Mohammed Shaikh, Z. & Dhar, S. Health literacy and self-reported hearing aid use in the health and retirement study. *Ear Hear* **41**, 386–394 (2020). 31369472 10.1097/AUD.0000000000000770

[28] World Medical Association Declaration of Helsinki. Recommendations guiding physicians in biomedical research involving human subjects. *JAMA* **277**, 925–926 (1997). 9062334

[29] Ministry of Health. Law. Ethical guidelines for medical and health research involving human subjects provisional translation. https://www.mhlw.go.jp/file/06-Seisakujouhou-10600000-Daijinkanboukouseikagakuka/0000080278.pdf (2024).

[30] American Academy of Otolaryngology-Head and Neck Surgery Foundation, INC. Committee on HEARing and Equilibrium guidelines for the evaluation of hearing preservation in acoustic neuroma (vestibular schwannoma). *Otolaryngol. Head Neck Surg.* **113**, 179–180 (1995). 7675475 10.1016/S0194-5998(95)70101-X

[31] Bratt, G. W. & Sammeth, C. A. Clinical implications of prescriptive formulas for hearing aid selection. In *The Vanderbilt Hearing Aid Report II* (eds Studebaker, G., Bess, F. & Beck, L.) 23–33 (York Press, Parkton, MD, 1991).

[32] Amos, N. E. & Humes, L. E. Contribution of high frequencies to speech recognition in quiet and noise in listeners with varying degrees of high-frequency sensorineural hearing loss. *J. Speech Lang. Hear. Res.* **50**, 819–834 (2007). 17675588 10.1044/1092-4388(2007/057)

[33] Hogan, C. A. & Turner, C. W. High-frequency audibility: benefits for hearing-impaired listeners. *J. Acoust. Soc. Am.* **104**, 432–441 (1998). 9670535 10.1121/1.423247

[34] Sugishita, M. et al. The validity and reliability of the Japanese Version of the Mini-Mental State Examination (MMSE-J) with the original procedure of the Attention and Calculation Task (2001). *Jpn J. Cogn. Neurosci.* **20**, 91–110 (2018).

[35] Smith, A. S. *Symbol Digit Modalities Test: Manual* (Western Psychological Services, 1982).

[36] Bennett, D. A. et al. Natural history of mild cognitive impairment in older persons. *Neurology* **59**, 198–205 (2002). 12136057 10.1212/wnl.59.2.198

[37] Hoffmann, K. et al. Moderate-to-high intensity physical exercise in patients with Alzheimer’s disease: a randomized controlled trial. *J. Alzheimers Dis.* **50**, 443–453 (2016). 26682695 10.3233/JAD-150817

[38] Aschwanden, D., Sutin, A. R., Luchetti, M., Stephan, Y. & Terracciano, A. Personality and dementia risk in England and Australia. *GeroPsych* **33**, 197–208 (2020). 34326756 10.1024/1662-9647/a000241 PMC8318004

[39] Keramat, S. A., Lee, V., Patel, R., Hashmi, R. & Comans, T. Cognitive impairment and health-related quality of life amongst older Australians: evidence from a longitudinal investigation. *Qual. Life Res.* **32**, 2911–2924 (2023). 37289356 10.1007/s11136-023-03449-3 PMC10473991

